# *In Vivo* Assessment of Mitochondrial Dysfunction in Clinical Populations Using Near-Infrared Spectroscopy

**DOI:** 10.3389/fphys.2017.00689

**Published:** 2017-09-14

**Authors:** T. Bradley Willingham, Kevin K. McCully

**Affiliations:** Department of Kinesiology, University of Georgia Athens, GA, United States

**Keywords:** oxidative capacity, muscle, neurologic disease, cardiovascular disease, optical spectroscopy

## Abstract

The ability to sustain submaximal exercise is largely dependent on the oxidative capacity of mitochondria within skeletal muscle, and impairments in oxidative metabolism have been implicated in many neurologic and cardiovascular pathologies. Here we review studies which have demonstrated the utility of Near-infrared spectroscopy (NIRS) as a method of evaluating of skeletal muscle mitochondrial dysfunction in clinical human populations. NIRS has been previously used to noninvasively measure tissue oxygen saturation, but recent studies have demonstrated the utility of NIRS as a method of evaluating skeletal muscle oxidative capacity using post-exercise recovery kinetics of oxygen metabolism. In comparison to historical methods of measuring muscle metabolic dysfunction *in vivo*, NIRS provides a more versatile and economical method of evaluating mitochondrial oxidative capacity in humans. These advantages generate great potential for the clinical applicability of NIRS as a means of evaluating muscle dysfunction in clinical populations.

## Introduction

Mitochondria serve critical roles in bioenergetics and cellular signaling. Specifically, mitochondria have been widely recognized for their ability to produce cellular free energy in the form of adenosine triphosphate (ATP) through oxidative phosphorylation. Oxidative phosphorylation is an oxygen-dependent biochemical process which converts biological fuels to ATP and functions as the primary mechanism of energy production at rest and during aerobic exercise. Skeletal muscle energy demands can increase 100-fold during exercise, and the ability to sustain submaximal exercise is largely dependent on the production of ATP via mitochondrial oxidative phosphorylation (Holloszy, 1967; Weibel and Hoppeler, 2005). Thus, muscle fibers containing high mitochondrial content are more resistant to the development of fatigue compared to muscle fibers with lower mitochondrial content (Pette, 1985; Casey et al., 1996). Mitochondrial biogenesis can be induced in skeletal muscle by stimuli such as repeated muscle contractions, and increases in mitochondrial content and function contribute to aerobic adaptations observed during exercise training (Ryan et al., 2013b; Hood et al., 2015; Navas-Enamorado et al., 2017). Alternatively, muscle disuse and pathology can result in mitochondrial dysfunction, and declines in mitochondrial content and/or function have been implicated in symptoms associated many neurological and cardiovascular diseases (Kent-Braun et al., 1994a, 1997; Vorgerd et al., 2000; Morino et al., 2005; Koves et al., 2008; McCully et al., 2011). Evaluating the role of skeletal muscle mitochondria dysfunction in the progression of pathology requires the development of clinically relevant assessments of mitochondria capacity. The present review will highlight recent studies related to *in vivo* assessments of skeletal muscle mitochondrial dysfunction in clinical human populations and address the potential for clinical translation of the findings.

Skeletal muscle mitochondria in humans has been historically evaluated using muscle tissue obtained from muscle biopsies, and many *in vitro* techniques have been employed to sequence mitochondrial DNA (mtDNA), quantify mitochondrial content, and evaluate enzyme activity levels in excised tissue samples. Indeed, these techniques provide important information related to specific mtDNA mutations and mitochondrial enzyme deficiencies, and therefore, play a critical role in the diagnosis and characterization of various mitochondrial diseases (Gorman et al., 2015; Karkucinska-Wieckowska et al., 2015; Nafisinia et al., 2017). However, sequencing mtDNA and measuring mitochondrial enzymes do not directly evaluate mitochondrial function, or the capacity of mitochondria to perform oxidative phosphorylation. Mitochondrial function is typically evaluated *in vitro* by measuring rates of oxygen consumption in permeabilized muscle fibers or isolated mitochondria using polargraphic oxygen sensors and phosphorescent oxygen-sensitive probes (Pesta and Gnaiger, 2012; Silva and Oliveira, 2012; Czajka and Malik, 2016). Changes in cellular NADH redox status and mitochondrial membrane potential can also be measured using fluorescence spectroscopy and have been reported as indices of mitochondrial function (Glancy et al., 2013). While *in vitro* assessments of mitochondrial function have many advantages, including the capacity to distinguish respiratory steady-states (Pesta and Gnaiger, 2012), these measurements are limited in their ability to reflect physiological mitochondrial function. For example, mitochondria are structured in an interconnected reticulum within skeletal muscle, and it has been recently demonstrated that the morphology of this reticulum facilitates energy distribution within the cell (Glancy et al., 2015; Patel et al., 2016). The structure of the reticulum is compromised in the *in vitro* preparation of the mitochondria and may influence measures of respiration. Furthermore, isolated mitochondria and permeabilized muscle fibers are expose to oxygen levels and temperatures that can differ from the natural physiological environment of the mitochondrion. Evaluating mitochondrial function *in vivo* can provide integrative measurements of oxidative capacity which include physiological temperatures, endogenous oxygen delivery systems, and preservation of the mitochondrial reticulum.

Previous assessments of mitochondrial function *in vivo* have employed indirect measures of oxidative capacity. Measures of indirect calorimetry using open-circuit spirometry have been broadly applied to quantify aerobic metabolism in humans, and studies have reported maximal whole body oxygen consumption rate and onset kinetics during exercise as measures of oxidative capacity (Bruce et al., 1973; Hansen et al., 2014). However, measures of oxygen consumption from open circuit spirometry are influenced by the cardiovascular, neurological, and skeletal muscle systems, and there is much deliberation surrounding the interpretation of these measures (Saltin and Strange, 1992; Wagner, 1996; Grassi, 2006). 31P MRS can be used as a muscle-specific alternative to measuring oxidative capacity by evaluating the recovery of phosphocreatine following a brief bout exercise. Since the regeneration of PCr is dependent on ATP production from aerobic metabolism, the rate of PCr recovery following exercise is indicative of the muscle's mitochondrial oxidative capacity (Chance et al., 1986; Forbes et al., 2009). While 31P MRS is a valid method of measuring mitochondrial function *in vivo*, the efficacy of this methodology is restricted by the cost and accessibility of magnets. More recent studies have demonstrated that near-infrared spectroscopy (NIRS) can be used in a similar manner to measure the recovery of oxygen metabolism following a brief bout exercise as an assessment of mitochondrial oxidative capacity (Ryan et al., 2012, 2013c, 2014a). The lower cost and portability of NIRS makes the methodology more readily available to researchers and healthcare professionals and increases the potential for integration into clinical practice.

## Near-infrared spectroscopy

**Figure 1:**
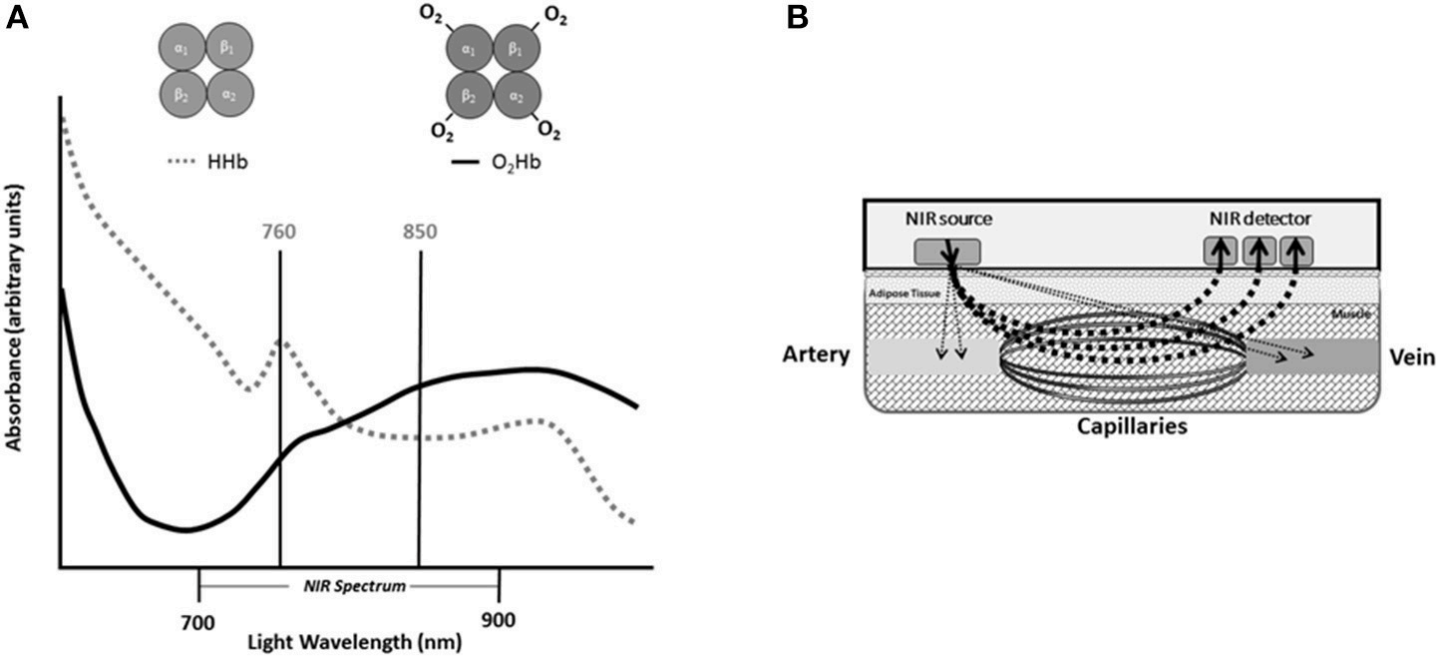
**(A)** Absorbance spectra for oxygenated hemoglobin (O2Hb) and deoxygenated hemoglobin (HHb) within the near infrared (NIR) spectrum of light. **(B)** Diagram of near infrared probe in human tissue.

**Figure 2:**
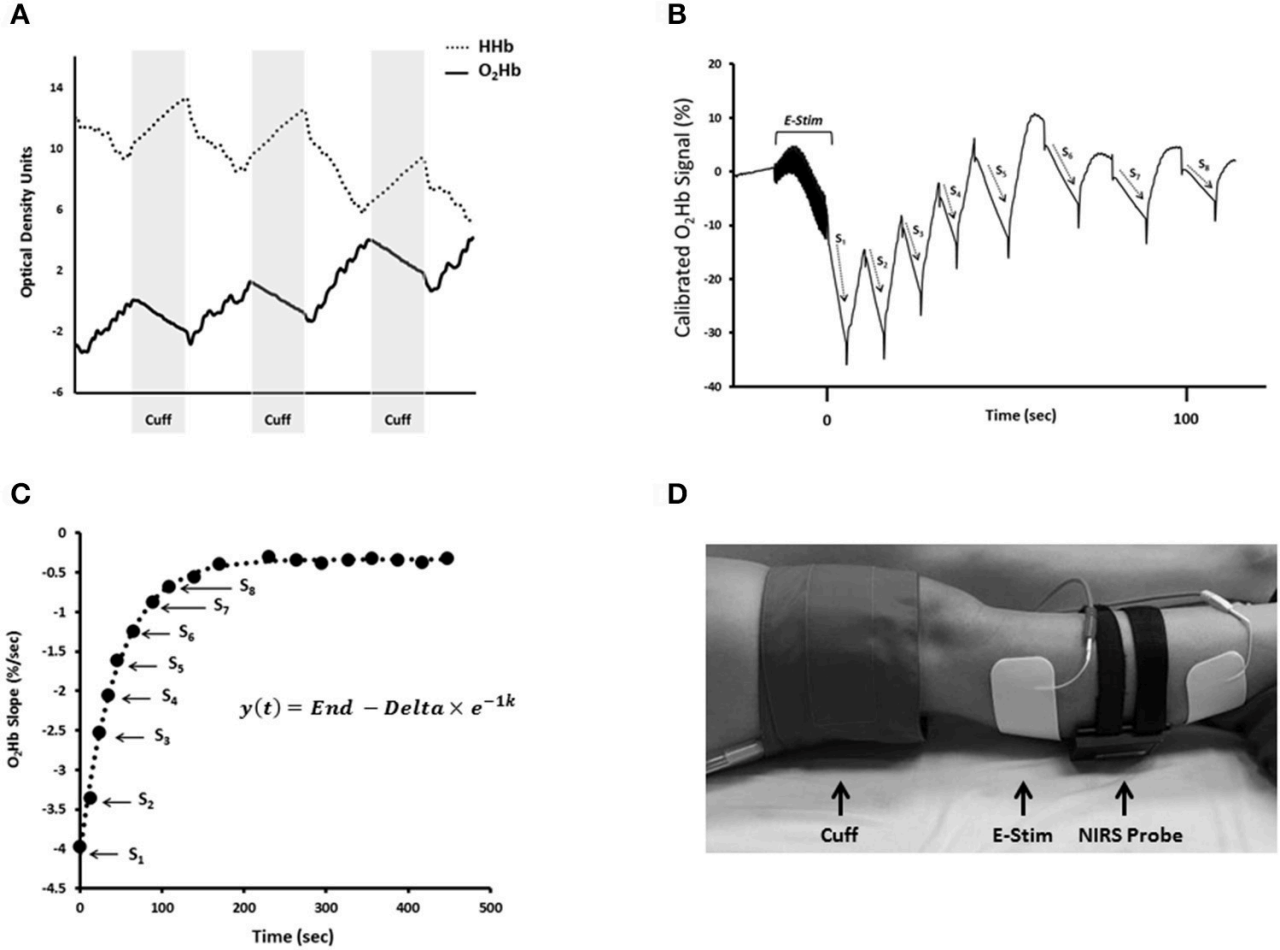
**(A)** Changes in oxygenated hemoglobin (O2Hb) and deoxygenated hemoglobin (HHb) during periods of cuff-induced ischemia. **(B)** NIRS O2Hb kinetics during a series of arterial occlusions following 15 s electrical stimulation (E-Stim). **(C)** Slope values from NIRS O2Hb recovery kinetics plotted over time and fit to exponential equation. In this equation, *y* is the relative rate of oxygen metabolism, *End* is the rate of oxygen metabolism at the end of exercise (S_1_), and Δ is the difference between the rates of resting oxygen metabolism and *End*. The rate constant, *k*, is used an index of muscle mitochondrial capacity. **(D)** NIRS set up for assessment of mitochondrial capacity in the gastrocnemius.

Since NIRS was first described by Frans Jobsis in 1977, the technique has been well established as a noninvasive method of measuring tissue oxygenation, blood flow, and metabolism (Jobsis, 1977). NIRS leverages the light absorbance properties of hemoglobin to measure changes in tissue oxygen saturation *in vivo* (Figure 1A). The longer wavelengths of near-infrared light (700–900 nm) experience less scattering and absorption than visible light, and allow the light to easily penetrate living tissues (Figure 1B; Jobsis, 1977). In human tissue, near-infrared light is primarily absorbed by hemoglobin and myoglobin, and the specific wavelength of light absorbed by the chromophore is dependent on the oxygenation status of hemoglobin/myoglobin (Jobsis, 1977). Therefore, changes in near-infrared light absorption can be used as indices of oxygen kinetics. The many applications of NIRS have been reviewed elsewhere in detail (Grassi and Quaresima, 2016). The present review will focus on the application of NIRS to evaluate mitochondrial oxidative capacity as first described by Ryan and McCully in 2012 (Ryan et al., 2012). Changes in NIRS signals during periods of ischemia can be used to measure the metabolism of oxygen in skeletal muscle (Figure 2A; Hamaoka et al., 1996; McCully et al., 2011; Ryan et al., 2012). The metabolism of oxygen as measured by NIRS reflects the reduction of molecular oxygen by cytochrome C oxidase (Complex IV) of the mitochondrial electron transport chain (ETC) during oxidative phosphorylation. Thus, the rate of oxygen metabolism increases as electron flux through the ETC increases during oxidative phosphorylation (Willis et al., 2016). Similar to 31P MRS, NIRS measures of mitochondrial oxidative capacity are based on the relationship between oxidative phosphorylation and PCr recovery following exercise (Paganini et al., 1997; Forbes et al., 2009). In brief, increases in the rate of oxygen metabolism as measured by NIRS during periods of ischemia following exercise reflect the increases in cellular respiration required to restore PCr in the muscle (Paganini et al., 1997; Ryan et al., 2013c). Thus, the recovery of muscle metabolic rate following exercise can be measured using a series of ischemic periods performed after a brief bout of exercise (Figure 2B). The rates of oxygen metabolism during the series of post-exercise cuffs can be fitted to an exponential function based on previously establish models (Mahler, 1985; Meyer, 1988; Paganini et al., 1997) of metabolic recovery kinetics: *y*(*t*) = *End* − Δ × *e*^−*kt*^ (Figure 2C). In this equation, *End* is the rate of oxygen metabolism at the end of exercise, and Δ is the difference in rate of oxygen metabolism between rest and the end of exercise. The rate constant, *k*, is calculated and used as an index of muscle mitochondrial capacity. Recovery rates of oxygen kinetics using this protocol express strong agreeability with measures of PCr recovery as measured by 31P MRS and maximal oxygen consumption rates as measured by high-resolution respirometry (Ryan et al., 2013c, 2014a). In addition to cross-validation with established methods of measuring mitochondrial function, NIRS measures of oxidative capacity also have suitable reproducibility (Southern et al., 2014). Notably, the optical spectroscopy, ischemia, and exercise components of the protocol are noninvasive and may be performed in either a clinic or laboratory setting (Figure 2D). Ischemia can be induced using a rapid pneumatic cuff system by inflating the cuff to a suprasystolic pressure proximal to the area of interrogation (Figure 2D). While the exercise may be performed using voluntary muscle contractions, surface neuromuscular electrical stimulation (E-Stim) can be used to provide further control over the intensity, frequency, and duration of muscle contractions (Figure 2D).

Studies have demonstrated the ability of NIRS to evaluate mitochondrial oxidative capacity in various muscles and as well as exercise-induced adaptations in muscle mitochondrial function (Ryan et al., 2013b). The more impactful application of NIRS, however, is the assessment of mitochondrial dysfunction in clinical populations. There has been increasing interests in the ability of NIRS to quickly and noninvasively measure muscle mitochondrial function in clinical populations that may be affected by declines in oxidative capacity (Erickson et al., 2013, 2015, 2016; Bossie et al., 2016). NIRS can evaluate mitochondrial function in various muscles affected by pathology without imposing the burden of muscle biopsies, performing rigorous exercise tests, or even removing patients from their treatment facility. In addition, NIRS also provides a method of evaluating the effectiveness of therapeutic interventions targeting muscle mitochondrial function (Erickson et al., 2016). Several recent studies have employed this methodology to evaluate mitochondrial function in various clinical populations, and the findings are providing novel insight into pathophysiology and rehabilitation interventions (McCully et al., 2011; Erickson et al., 2013, 2015, 2016; Ryan et al., 2013d, 2014b; Bossie et al., 2016).

### Neurological injury and disease

#### Spinal cord injury

Spinal Cord Injury (SCI) often results in deactivation of skeletal muscle below the level of injury. The loss of chronic skeletal muscle activation can lead to muscle atrophy and changes in muscle phenotype which may impede the rehabilitation processes and increase cardiometabolic risk (Shields, 1995; Castro et al., 1999, 2000; Gerrits et al., 1999; Stein et al., 2002; Elder et al., 2004; Shields et al., 2006; Banerjea et al., 2008; Pelletier and Hicks, 2011). The physiological changes in skeletal muscle following a SCI have been historically evaluated using muscle biopsies from the affected (paralyzed) limbs, and numerous studies have demonstrated that skeletal muscle transitions to a slow twitch, glycolytic phenotype following a SCI (Shields, 1995; Castro et al., 1999, 2000; Gerrits et al., 1999; Pelletier and Hicks, 2011). Accordingly, histochemical assays of skeletal muscle in person with SCI have found decreased mitochondrial content and a ~50% reduction in oxidative enzyme activity compared to controls (Castro et al., 1999; Chilibeck et al., 1999; Gregory et al., 2003). *In vivo* studies using 31P MRS have also reported a ~50% reduction in muscle oxidative capacity in persons with SCI (McCully et al., 2011). Erickson et al. first used NIRS to evaluate mitochondrial oxidative capacity in the vastus lateralis of nine persons with motor complete SCI (Erickson et al., 2013). In agreement with 31P MRS studies, the authors reported a 50–60% reduction mitochondrial oxidative capacity as measured by NIRS in persons with SCI compared to able bodied controls (McCully et al., 2011; Erickson et al., 2013).

Skeletal muscle in persons SCI has also demonstrated considerable plasticity (Shields et al., 2006; Shields and Dudley-Javoroski, 2007; Petrie et al., 2014a,b; Woelfel et al., 2016). Specifically, electrical stimulation has shown to have robust effects the function and phenotype of paralyzed muscle tissue (Dudley-Javoroski and Shields, 2008). Studies have found that electrical stimulation training of muscle can result in increased muscle endurance, preservation of slow muscle fibers, and increases in oxidative enzyme activity and gene expression (Martin et al., 1992; Andersen et al., 1996; Mohr et al., 1997; Gerrits et al., 2000; Shields and Dudley-Javoroski, 2006; Petrie et al., 2014b). These findings indicate that the improvements in muscle function following electrical stimulation training may be mediated by increases in mitochondrial capacity and highlight the need for clinical evaluation tools to monitor skeletal muscle function in this population. Ryan et al. evaluated the effect of a progressive electrical stimulation training program on the mitochondrial oxidative capacity of vastus lateralis using NIRS in a single individual with a chronic C5 motor complete SCI (Ryan et al., 2013d). After 24 weeks of training, oxidative capacity as measured by NIRS increased nearly 3-fold. Notably, the post-training oxidative capacity of the paralyzed muscle was comparable to previously reported measures from able bodied persons (Erickson et al., 2013; Ryan et al., 2013d). In a more recent study of fourteen persons with SCI performing a similar progressive electrical stimulation training protocol, oxidative capacity of the vastus lateralis as measured by NIRS increased ~2-fold on average (Erickson et al., 2016). These studies demonstrate the usefulness of NIRS as a method of evaluating the effects of therapeutic interventions that does not require invasive muscle biopsies or expense of MRS.

#### Multiple sclerosis

Multiple sclerosis (MS) is an autoimmune disease which causes degradation of myelin and axonal damage in the central nervous system (CNS). The demyelination of axons impairs neural transmission in the CNS which can impede voluntary muscle activation and motor control (Kent-Braun and Le Blanc, 1996; Higginson et al., 2006; Andreasen et al., 2009; Newland et al., 2012). Similar to SCI, studies have shown that MS is also associated with changes within skeletal muscle which are secondary to primary deficits in the CNS (Kent-Braun et al., 1997; Castro et al., 1998; Garner and Widrick, 2003; Carroll et al., 2005). A 40% reduction in succinate dehydrogenase activity and 30% lower capacity to oxidize NADH have been reported in skeletal muscle from persons MS compared to controls (Costill et al., 1976; Kent-Braun et al., 1997; Kumleh et al., 2006). However, findings related to alterations in contractile proteins have varied (Costill et al., 1976; Kent-Braun et al., 1997). Skeletal muscle oxidative metabolism in person with MS has also been measured *in vivo* using 31P MRS and NIRS (Kent-Braun et al., 1994a,b; Malagoni et al., 2013). Kent-Braun et al. reported a 46% reduction in PCr recovery following exercise in the tibialis anterior muscle of persons with MS compared to controls, indicating significantly impaired oxidative metabolism (Kent-Braun et al., 1994a). Studies using NIRS to measure fractional oxygen consumption in the lower extremity muscles have found resting metabolism to either be increased or no different in persons compared to controls (Malagoni et al., 2013). However, these methodologies can be influenced by subcutaneous adipose tissue thickness and total hemoglobin content and may not reflect mitochondrial oxidative capacity. Harp et al. recently employed the NIRS recovery test to measure oxidative capacity in the gastrocnemius of 16 persons with MS (Harp et al., 2016). Oxidative capacity as measured by NIRS was found to be 40% lower in persons with MS compared to controls (Harp et al., 2016). These findings are consistent with *in vitro* studies of muscle biopsies and those using 31P MRS (Kent-Braun et al., 1994a,b).

Previous studies have suggested that oxidative capacity may be related to deficits in physical function in persons with MS. For example, Hasen et al. found oxygen onset kinetics during exercise to be strongly related to walking ability (Hansen et al., 2014). A previous study also found significant bilateral differences in peak VO2 from isolated limb cycling, and the magnitude of differences between limb was inversely related to walking ability (Larson et al., 2013). However, measures of whole body oxygen consumption is limited in its ability to measure muscle specific oxidative capacity, particularly in populations where central nervous and cardiovascular systems may be affected (Grassi, 2006). When evaluating asymmetry in muscle-specific oxidative capacity and relation to walking function, Harp et al. did not find bilateral differences in NIRS measures of mitochondrial capacity or any relationship to walking speed (Harp et al., 2016). Thus, the role of muscle oxidative dysfunction in the development of functional deficits, specifically walking ability, is unclear and warrants further investigation.

#### Amyotrophic lateral sclerosis

Amyotrophic Lateral Sclerosis (ALS) is a progressive neurological disease characterized by the degradation of motor neurons in the CNS. While the damage to cerebral, spinal, and peripheral neurons has been implicated in the progression of ALS, there is increasing evidence suggesting that skeletal muscle mitochondria are also directly affected by the pathology (Leclerc et al., 2001; Krasnianski et al., 2005; Artuso et al., 2013; Luo et al., 2013; Al-Sarraj et al., 2014). Studies in humans and mouse models of ALS have found mitochondrial DNA mutations and disruption of mitochondrial fusion/fission kinetics (Bowling et al., 1993; Artuso et al., 2013; Luo et al., 2013). However, *in vivo* measures of mitochondrial oxidative capacity using 31P MRS have not identified abnormalities in mitochondrial function in person with ALS (Grehl et al., 2007). Ryan et al. employed both 31P MRS and NIRS techniques in seven persons with ALS and four age-matched controls (Ryan et al., 2014b). In agreement with previous studies, no significant difference in measures of oxidative capacity was found between groups using either *in vivo* methodology. Moreover, the authors reported strong agreeability between the *in vivo* methodologies, indicating that both oxygen utilization and ATP production in skeletal muscle were both unaffected by the pathology.

### Cardiovascular an respiratory disease

#### Heart failure

Heart Failure (HF) is a progressive condition associated with poor quality of life and multiple comorbidities. In addition, reduced exercise capacity is common in persons with HF, but the link between the pathophysiology of HF and exercise intolerance is unclear (Franciosa et al., 1981). Studies evaluating exercise intolerance in persons with HF have reported that deficiencies in cardiac contractility, cell signaling, and skeletal muscle function may all contribute to exercise intolerance in this population (Mancini et al., 1989; Sullivan et al., 1990; Fukushima et al., 2013; Kitzman et al., 2014; Feng et al., 2015). Specifically, some evidence suggests that muscle mitochondrial function may be related to reduced muscle oxidative capacity in persons with HF (Sullivan et al., 1990; Arnolda et al., 1991; Mancini et al., 1992; Chati et al., 1996; Simonini et al., 1996; Toth et al., 2012). For example, *in vitro* studies using muscle biopsies have reported a ~40% reduction in mitochondrial enzyme activity in persons with HF compared to control (Sullivan et al., 1990). Furthermore, *in vivo* studies using 31P MRS have reported a ~50% reduction in oxidative capacity of the gastrocnemius muscle (Mancini et al., 1989). Southern et al. used NIRS to evaluate muscle mitochondrial capacity in the forearm of 16 participants with HF and found that recovery rate constants were ~20% lower in participants with HF compared to controls (Southern et al., 2015). While these findings suggest that muscle metabolism is negatively affected by HF, the extent of impairment was not as large as previously reported in the calf (Mancini et al., 1989). The discrepancy between these measures may be driven by differences in the utilization of muscle groups. Studies have shown that persons with HF have decreased physical activity, so it is possible that the greater deficit in oxidative capacity observe in the lower limb muscles using 31P MRS may be more closely related decreased muscle use, whereas the forearm muscle may be less influenced by physical activity levels (Mancini et al., 1989; Chati et al., 1996).

In the study by Southern et al., a subgroup of 7 participants with HF and 5 controls also completed 4 weeks of forearm endurance training to evaluate exercise-induced adaptations of muscle mitochondrial capacity in this population (Southern et al., 2015). Interestingly, mitochondrial capacity was unaffected by training in participants with HF despite a ~50% improvement in the control group. These results suggest that mitochondrial adaptations to exercise may be impaired in persons with HF. However, further research is warranted as the effect of pharmacological interactions with exercise in persons with HF remains unclear.

#### Cystic fibrosis

Cystic Fibrosis (CF) is an autosomal recessive genetic disease which results in dysfunction of the CF transmembrane conductance regulator (CFTR) protein. CFTR is a chloride ion channel that plays a critical role in the regulation of mucus in the airway, and persons with CF experience deficiencies in clearance which can result in chronic bacterial infections and inflammation (Kumar et al., 2014). CF is also associated with reduce exercise tolerance, and studies have found that exercise capacity (VO_2Peak_) is associated with survival (Nixon et al., 1992; Pianosi et al., 2005a). Importantly, the reported relationship between VO_2Peak_ and mortality was independent of respiratory function (FEV1), suggesting that other factors may be involved (Pianosi et al., 2005b). Several studies have shown that persons with CF have abnormalities in skeletal muscle strength and metabolic capacity (Shapiro, 1989; Wells et al., 2011). Specifically, Wells et al. reported a 29% reduction in PCr recovery kinetics in the vastus lateralis of persons with CF compared to controls, indicating muscle-specific impairments oxidative capacity (Wells et al., 2011). Erickson et al. evaluated muscle mitochondrial capacity in the vastus lateralis of 13 persons with CF and 16 healthy controls using NIRS (Erickson et al., 2015). This study reported a ~15% reduction in muscle mitochondrial capacity in persons with CF compared to healthy controls (Erickson et al., 2015). Furthermore, Erickson et al. found no significant relationship between FEV1 and NIRS measures of muscle mitochondrial capacity, which is in agreement with previous studies evaluating the relationship between whole body exercise capacity and mortality. These findings suggest that skeletal muscle dysfunction may independently contribute to exercise intolerance in persons with CF. However, future studies are needed to evaluate the role of physical activity in the decline in mitochondrial capacity in persons with CF and the utility of NIRS measures of mitochondrial capacity as a biomarker of exercise tolerance in this population.

#### Chronic obstructive pulmonary disease

Chronic Obstructive Pulmonary Disease (COPD) is progressive condition characterized by reduced airflow during pulmonary ventilation resulting from thickening of airways, reduced alveoli elasticity, and chronic inflammation (MacNee, 2005). COPD is associated with a host of comorbidities, including muscle dysfunction and reduced exercise capacity (Ries et al., 1995; Remels et al., 2013; Negewo et al., 2015). Muscle dysfunction in COPD is characterized by cachexia, decreased strength, and changes in muscle metabolic properties which favor a more glycolytic phenotype (Maltais et al., 2000a,b; Gosker et al., 2007b; Remels et al., 2013). Studies using muscle tissues from the vastus lateralis have reported ~50% lower in mitochondrial content and ~25% lower citrate synthase activity in persons with COPD compared to controls (Maltais et al., 2000a,b; Gosker et al., 2007a). More recent *in vivo* studies using 31P MRS to measure mitochondrial capacity indicate that deficits in oxidative function in persons with COPD may be more closely related to physical activity (Shields et al., 2015). Shields et al. measured PCr recovery in both the quadriceps and biceps brachii muscles and found that recovery times were only slower in the lower extremity muscles of persons with COPD compared to the controls (Shields et al., 2015). Comparably, Layec et al. found no difference in PCr recovery in the lower limb muscles between persons with COPD and controls with similar physical activity levels (Layec et al., 2017). Adami et. al. used NIRS to evaluate mitochondrial capacity in the gastrocnemius muscle of 28 individuals with COPD and 28 controls (Adami et al., 2017). The authors found that mitochondrial capacity was 25% lower in the gastrocnemius of persons with COPD compared to the control group. While these findings are in contrast to previous studies using 31P MRS, the participants in the control group were significantly younger in age, and both groups had mitochondrial capacity lower than values previously reported for even younger individuals (ages 18–27) (Adami et al., 2017). Therefore, the differences between groups could be a result of differences in age, but more research is needed to determine the effect of aging on muscle mitochondrial function independent of physical activity. Adami et al. also found that NIRS measures of mitochondrial capacity had reproducibility comparable to values reported in controls (coefficient of variation ~10%; Interclass correlation coefficient = ~0.9), indicating that NIRS may be used as a reliable method of measuring muscle mitochondrial capacity in persons with COPD and older adults.

### Mitochondrial disease

#### Friedreich ataxia

Friedreich Ataxia (FRDA) is a mitochondrial disease caused by a genetic mutation of the FRDA gene. Alterations of the FRDA gene sequence can disrupt production of the mitochondrial protein frataxin, and studies have shown that persons with FRDA have lower levels of frataxin (Priller et al., 1997; Bradley et al., 2000). Although the exact role of frataxin in mitochondrial function is unclear, studies using genetic yeast models indicate that the protein is involved in mitochondrial iron regulation, respiration, and mitochondrial DNA repair (Bradley et al., 2000). *In vitro* studies of respiratory chain function in humans with FRDA have found 84% reduction in complex I and 77% complex III activity in cardiac muscle, but no significant reduction was found in skeletal muscle (Bradley et al., 2000). Alternatively, several studies using 31P MRS to evaluate mitochondrial function *in vivo* have found mitochondrial capacity of the calf muscle to be ~70% lower in persons with FRDA compared to controls (Lodi et al., 1999; Vorgerd et al., 2000). Similarly, Lynch et. al. found that post-exercise recovery of oxygen saturation was ~50% slower in the medial gastrocnemius of persons with FRDA compared to controls (Lynch et al., 2002). Bossie et al. measured oxidative capacity on the forearm muscle of 16 persons with FRDA using NIRS (Bossie et al., 2016). In contrast to studies in lower limbs, Bossie et al. did not find any difference in forearm muscle oxidative capacity in persons with FRDA compared to controls (Bossie et al., 2016). The lack of agreement among these studies may indicate that impairments in mitochondrial capacity in the lower limb may be more closely related to disuse rather than pathophysiology. However, NIRS measures of mitochondrial capacity in the forearm were inversely correlated with feelings of low energy, suggesting that mitochondrial function may be related to fatigue in persons with FRDA across the spectrum of symptom severity (Bossie et al., 2016). These findings lend support to the use of NIRS measures of mitochondrial capacity as measure of muscle dysfunction in persons with FRDA during interventions or over the course of disease progression.

Compared to the aforementioned pathologies associated with mitochondrial dysfunction, mitochondrial disease research is relatively limited with the medical treatment of mitochondrial disease only emerging within the last 25 years (Parikh et al., 2014, 2015). Thus, there is increasing awareness surrounding the challenges related to the diagnosis and treatment of patients with mitochondrial disease and the need for clinical assessments of mitochondrial dysfunction in this population. There are currently no physiological evaluation tools available for clinicians diagnosing and treating patients with mitochondrial disease, and the application of NIRS may offer a relevant, *in vivo* measure of mitochondrial function in this population. To date, Fredrich's ataxia is the only mitochondrial disease to be evaluated using NIRS (Bossie et al., 2016), and establishing the usefulness of NIRS in evaluating mitochondrial dysfunction in persons with mitochondrial disease warrants further investigation.

## Discussion

**Figure 3:**
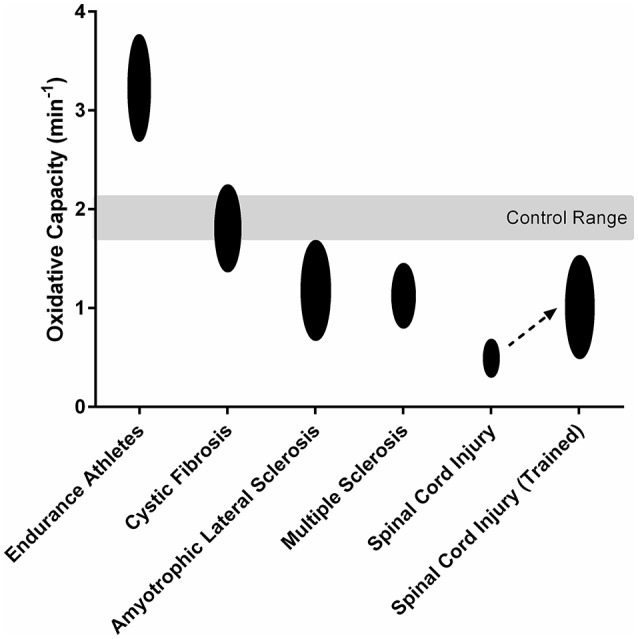
Mitochondrial oxidative capacity of the lower limb muscles in endurance athletes (Brizendine et al., 2013) and persons with cystic fibrosis (Erickson et al., 2015), amyotrophic lateral sclerosis (Ryan et al., 2014b), multiple sclerosis (Harp et al., 2016), and spinal cord injury (Erickson et al., 2016). Spinal Cord Injury (Trained) shows the 119% increase in mitochondrial oxidative capacity observed after 16 weeks electrical stimulation training (Erickson et al., 2016).

The assessment of mitochondrial function is important in the study of pathophysiology and efficacy of medical interventions in various clinical populations. Recent studies have demonstrated the utility of NIRS in the evaluation of mitochondrial dysfunction in persons with neurological, autoimmune, cardiovascular, and mitochondrial diseases (Figure 3; Erickson et al., 2013, 2016; Ryan et al., 2013d, 2014b; Southern et al., 2015; Bossie et al., 2016). Furthermore, NIRS measures of oxidative capacity have also been used to assess the effects of various therapeutic interventions targeting skeletal muscle dysfunction in clinical populations (Ryan et al., 2013d; Erickson et al., 2016). In summary, NIRS can provide a reliable and physiologically relevant assessment mitochondrial oxidative capacity in clinical populations.

Previous techniques used to measure muscle mitochondrial function, such as muscle biopsies and MRI, have several limitations in evaluating mitochondrial function in clinical populations, during interventions, and in characterizing changes over time. Muscle biopsies require invasive, surgical procedures that increase participant burden and limit the area of interrogation to a small (100–200 mg) tissue sample (Shanely et al., 2014). While NIRS is also limited to a small area of interrogation (2–6 cm^3^), the noninvasive nature of the technique permits multiple measurements to be performed in close proximity (Grassi and Quaresima, 2016). Furthermore, the depth of NIRS measures is determined by the distance between the light source and receiver (measurement depth = ~50% of the interoptode distance), which allows for control of measurement depth. Therefore, NIRS may be more applicable in characterizing mitochondrial capacity at multiple sites within a single muscle group or between muscles of variable tissue thickness. The capacity to evaluate multiple muscle groups may be particularly beneficial in populations where disuse of specific muscles (lower vs. upper extremities) may influence changes in metabolic phenotype. In addition, repeatability of NIRS measures provides an advantage when assessing the effect of interventions or characterizing changes over time. The injury from a biopsy may interfere with the ability to perform therapeutic interventions for 48–72 h, and multiple tissue samples must be obtained to characterize changes in mitochondrial function over time (Shanely et al., 2014). NIRS measures of mitochondrial capacity do not result in any injury or other prolonged changes in skeletal muscle function. Thus, NIRS measures can be obtained frequently throughout rehabilitation or pharmaceutical interventions as recently demonstrated in healthy and clinical populations (Ryan et al., 2013d,b; Erickson et al., 2016). While 31P MRS offers an alternative noninvasive approach for evaluating muscle mitochondrial function, MRI can be costly, and lack of mobility may limit accessibility to some individuals. NIRS devices are ~10% the cost of MRI, and NIRS measures can be acquired in a bedside manner without a high level of expertise. NIRS provides the affordability and portability needed to scale the technique into clinical practice.

Evaluation of metabolic capacity *in vivo* requires the activation of skeletal muscle. Previous studies using NIRS to evaluate the mitochondrial oxidative capacity of skeletal muscle have used voluntary and electrically induced muscle contractions, and both methods have demonstrated strong agreeability and similar reproducibility (Ryan et al., 2013a; Southern et al., 2014). Ergometers and resistance bands can be used to perform voluntary exercise, but the technique, activation patterns, and frequency of contractions may vary between participants (Southern et al., 2014). While variations in the intensity and duration of voluntary exercise can influence the initial metabolic rate, studies have shown that initial metabolic rate does not influence measures of mitochondrial capacity as NIRS recovery kinetics are exponential (Ryan et al., 2013a). However, high force, tetanic contractions produced during voluntary exercise may limit oxygen delivery and influence measures of metabolism. The use of electrical stimulation provides many advantages over voluntary exercise. The use of low frequency, twitch electrical stimulation provides a sufficient metabolic stimulus without requiring voluntary activation of skeletal muscle or depleting oxygen saturation. Previous studies have used NIRS in conjunction with electrical stimulation to evaluate muscle mitochondrial capacity in populations where voluntary activation is affected by injury or pathology (Erickson et al., 2013, 2016; Ryan et al., 2013d; Harp et al., 2016). Furthermore, low frequency twitch electrical stimulation produce low force contractions which may reduce risk musculoskeletal injury (Hartkopp et al., 1998). In healthy populations, electrical stimulation also provides the advantage of controlling the intensity, frequency, and duration of the exercise as well as the area of muscle fiber recruitment.

One of the challenges of optical spectroscopy is accounting for the scattering of light. Although NIR light can easily penetrate human tissue, the subcutaneous adipose tissue between the skin and the muscle can increase scattering and influence signal intensity (Homma et al., 1996; van Beekvelt et al., 2001). Specifically, studies have shown that subcutaneous adipose tissue thickness (ATT) can confound NIRS measures of oxygen consumption and blood flow (van Beekvelt et al., 2001). Therefore, it is critical to account ATT when measure mitochondrial capacity using NIRS, particularly in clinical populations where ATT may be greater. The “physiological calibration” has been developed as a method of accounting for the effects of ATT on signal intensity by calibrating the NIRS optical density units to a percentage of oxygen saturation (Hamaoka et al., 1996; Ryan et al., 2012). By employing the physiological calibration, metabolism can be measured as a change in percentage of the physiological range and allows for comparisons between muscles with different ATT as well as between individuals. In addition, ATT can also limit the ability of the NIR light to reach the muscle tissue if the subcutaneous fat layer is too thick to penetrate by widening the distance between the NIR transmitter and receiver, and therefore, may pose challenges in clinical populations associated with increased adiposity.

The applications of NIRS in clinical populations has provided novel information related to mitochondrial dysfunction associated with various pathologies. Notwithstanding, it should be considered that NIRS provides an integrated measure of mitochondrial oxidative capacity. NIRS measures do not provide insights into the potential mechanism(s) responsible for changes in mitochondrial capacity. For example, NIRS cannot identify changes in mitochondrial volume, substrate utilization, or dysfunction within the electron transport system. Indeed, the mechanisms underlying mitochondrial dysfunction are different depending on the pathology, and future studies may be strengthened by combining NIRS with *in vitro* methodologies or further developing optical techniques to evaluate electron transport within the mitochondria *in vivo*.

## Conclusion

Recent studies have demonstrated the utility of NIRS in the evaluation of mitochondrial dysfunction in persons with neurological, autoimmune, cardiovascular, and mitochondrial diseases. NIRS provides an affordable, versatile, and noninvasive technique for evaluating mitochondrial oxidative capacity of skeletal muscle in clinical populations.

## Author contributions

TW performed literature review and composed manuscript. KM designed and revised manuscript.

### Conflict of interest statement

KM is the president of Infrared RX. The other author declares that the research was conducted in the absence of any commercial or financial relationships that could be construed as a potential conflict of interest.
